# Does black phosphorus hold potential to overcome graphene oxide? A comparative review of their promising application for cancer therapy

**DOI:** 10.1039/d1na00203a

**Published:** 2021-05-28

**Authors:** Amalia Ruiz, Cristina Martín, Giacomo Reina

**Affiliations:** School of Pharmacy, Queen's University Belfast Belfast BT9 7BL UK; Dpto. de Bioingeniería en Ingeniería Aeroespacial, Universidad Carlos III de Madrid Avda. de la Universidad, 30. 28911 Leganés Madrid Spain; Strasbourg 67000 France giacomo.reina@gmail.com

## Abstract

Although graphene oxide (GO) is leading the way in the biomedical field of 2D materials, nanosized black phosphorus (NBP) has recently come to attention for use in this challenging field. A direct comparison between these two materials, in this context, has never been described. Therefore, in this mini-review, we will critically compare the applications of NBP and GO in cancer therapy. Material functionalisation, biodegradation by design, phototherapy and immunotherapy will be summarised. This work aims to inspire researchers in designing the next generation of safe NBP platforms for cancer treatment, taking advantage of the vast experience gained with GO.

## Abstract 2

In this mini-review, we will critically compare the applications of NBP and GO in cancer therapy focusing on material functionalization, biodegradation by design, phototherapy and immunotherapy.

## Graphical abstract

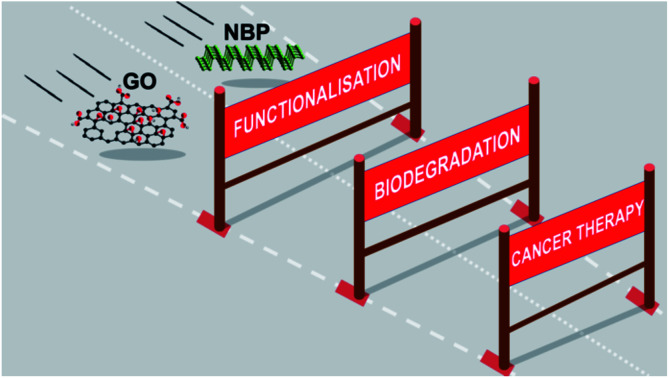

## 1 Introduction

Graphene oxide (GO) is certainly the most studied 2D material for biomedical applications. In particular, GO has been extensively studied in cancer therapy, where it has been used for targeting, drug delivery, and photo- and immunotherapy.^1,2^ More recently, nanosized black phosphorus (NBP) has attracted attention as an important tool in nanomedicine. The first results reported show that NBP is biocompatible, active in drug delivery, and bears phototherapeutic activity *per se*, making this nanomaterial a rising star in nanomedicine.^3^

Although GO and NBP are both 2D-materials applied in cancer treatment, there are several differences between these two nanostructures. In this mini-review, we will compare the strategies proposed for the preparation of GO and NBP-based platforms for cancer therapy. For the sake of clarity, no distinction based on the size of NBP (*e.g.*, dots or flakes) will be done in this review. Synthesis and functionalisation, biodegradation by-design, and application in photo-/immunotherapy will be presented and critically discussed, putting emphasis on the most recent results reported in the literature. Additionally, the limits and new challenges of both GO and NBP will be explored. With this contribution we hope to genuinely inspire scientists working on the future applications of GO and NBP in cancer therapy.

## 2 Synthesis and functionalisation of NBP and GO

NBP and GO are two nanomaterials produced by top-down strategies. In particular, GO is produced by a graphite exfoliation/oxidation process and primarily the Hummers method. To date, different modifications on Hummers' method have been reported. In general, potassium permanganate and hydrogen peroxide are used.^4^ The mechanism of oxidation and exfoliation has been investigated showing the role of water, sulfuric acid and permanganate concentration.^5^

NBP has only recently been synthesized at the lab-scale.^3^ NBP can be prepared from the exfoliation of bulk black phosphorous in different solvents; the most common are *N*-methyl-2-pyrrolidone (NMP), *N*,*N*-dimethylformamide and DMSO. The exfoliation process can be conducted *via* sonication or through mechanical shearing.^3^

Synthetic protocols of GO and NBP are scalable, making them appealing for industrial production. However, some concerns should be addressed about safety and costs. Additionally, top-down methods always require post-synthetic processes. In particular, removal of unexfoliated material and size selection are obtained *via* differential centrifugation for both GO and NBP. Regarding GO, the oxidation step is strongly exothermal in the presence of acids. This process generates harmful side-products that need to be treated accordingly. More recently, refinement of exfoliation/oxidation strategies has been validated for large-scale production with low batch-to-batch variation.^6^ In this context, it has been reported that controlling the oxidation conditions produces a low-defect GO surface.^6,7^ Other methodologies, such as jet-milling or electrochemical exfoliation,^8^ have also been validated for high-quality GO production with low polydispersity. With respect to NBP, liquid exfoliation can be conducted at room temperature. However, bulk black phosphorus is quite expensive to produce, and due to its low stability in air, it must be carefully handled. Additionally, a few articles reported exfoliation in water,^9^ but most of the protocols require toxic solvents with a yield that never exceeds 50%, resulting in amounts that might only be sufficient for research purposes.^10^ New strategies for the production of NBP have been reported using red phosphorous (RP) as a starting material.^11^ RP is a much less expensive precursor but more prone to ignition at room temperature. RP has been converted to NBP *via* high energy ball milling using PEG (polyethylene glycol) as a capping agent^12^ or using single-pass catalytic conversion that allows continuous production with low wastes and a few further purification steps.^11^ Additionally, both GO and NBP can also be produced in the form of 0D materials (dots). These 0D nanostructures have spectroscopic properties, making them useful for imaging and photodynamic therapy. However, while GO quantum dots (composed of a highly defective C structure and should not be confused with graphene quantum dots)^13^ have been so far more applied in sensing and catalysis;^14,15^ BP dots have been studied much more and applied in cancer research, and so only NBP dots will be treated in this work.

**Fig. 1:**
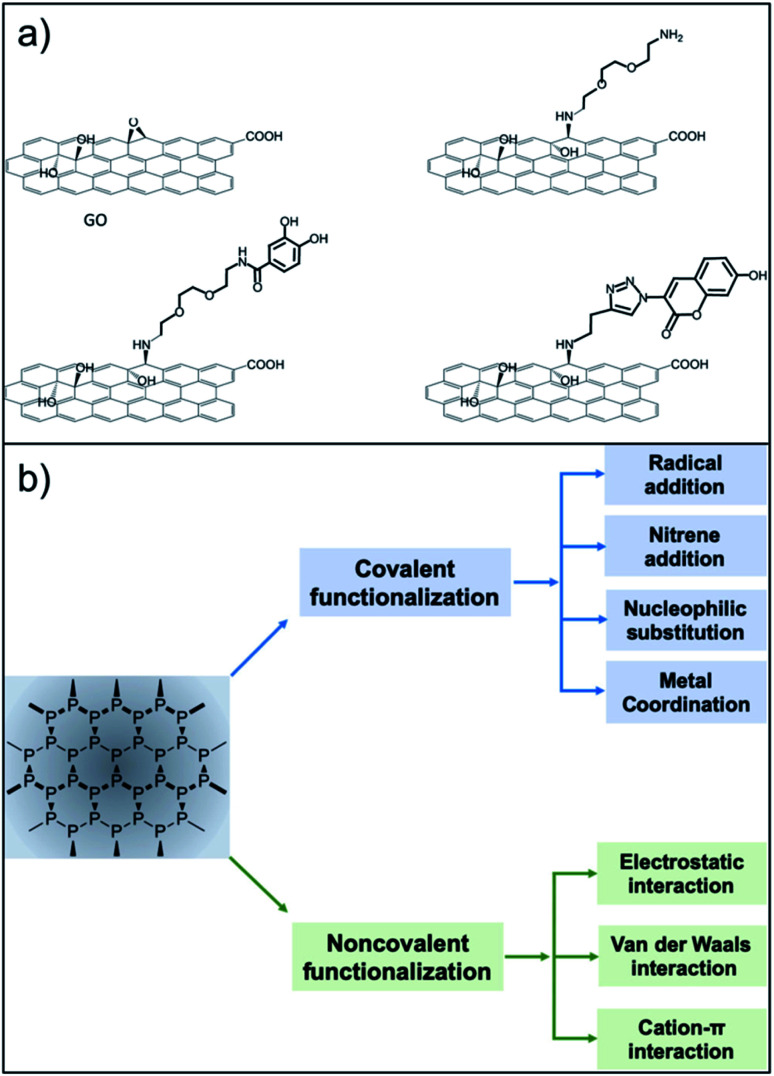
Functionalisation routes used for the preparation of GO and NPB nanoplatforms. (a) The versatile approach of the epoxide opening reaction with primary amines (reproduced from ref. 16 with permission from IOPscience, copyright Creative Commons Attribution 3.0 licence 2018). (b) Strategies for functionalisation of BP (reproduced from ref. 17 with permission from Wiley, copyright Creative Commons Attribution 3.0 licence 2019).

Surface functionalisation is essential for the preparation of efficient nanosized biomedical platforms facilitating the loading of drugs, targeting agents, dispersants or imaging probes into their formulation. In this context, both covalent and non-covalent approaches are used in order to maximize the final efficacy of the desired nanomaterial (Fig. 1).

Among all of the hard nanomaterials (*e.g.*, metallic or inorganic nanoparticles), GO chemistry is one of the most developed.^16^ GO is composed of the typical sp^2^ C honeycomb structure of graphene, where 30–50% of C is bound to an oxygenated functional group.^6^ Thus, GO chemistry includes sp^2^ C chemistry (Diels–Alder reactions, radical reactions, *etc.*) and the chemistry of the oxygenated functional groups.^2^ Additionally, the desired molecules can be easily adsorbed onto the GO surface through π–π stacking or electrostatic interactions. Regarding covalent functionalisation, amide formation (from GO carboxylic acids) is the most claimed functionalisation reaction. However, different studies have pointed out that, due to the low presence of carboxylic acids on the GO surface, this reaction has a low yield, while nucleophilic substitution (from GO epoxides) is the main product of GO with amines.^17,18^ This approach is very versatile, where amines or thiols can be used as nucleophiles.^19^ Additionally, alcohols placed on the GO surface can be targeted for its functionalisation using benzoquinone,^19^ silanes,^20^ and the Williamson reaction,^21^ and boronic esters can be formed from diols.^22^ GO is also a well-known material to improve the final properties (mechanical, electrical, *etc.*…) of GO-containing hydrogels.^23,24^ Guilbaud-Chéreau *et al.* recently reported protected amino acid-based hydrogels incorporating graphene oxide for NIR-triggered drug release applications.^25^

The surface chemistry of NBP is less developed.^26^ The relatively short shelf-life of BP under ambient conditions is attributed to its unstable bonding structure, where lone-pair electrons residing on the phosphorus atoms adsorb oxygen molecules, making NBP easily oxidised in air or water.^27,28^ NBP is a highly homogeneous material constituted only by phosphorus that presents some phosphate groups. Thus, it offers less possibilities for covalent functionalisation. Chemical routes refer to the surface modification by organic compounds such as the aryl diazonium ligand,^29^ octadecyltrichlorosilane^30^ or 1-butyl-3-methylimidazolium tetrafluoroborate,^31^ among others.^32,33^ At the moment, most of the functionalisation approaches deal with non-covalent chemistry, exploiting the electrostatic interaction between the negatively charged NBP and the selected molecule. In this context, biocompatible polymers such as amino-terminated polyethylene glycol,^34^ polylysine,^35^ polydopamine,^36^ poly(lactic-*co*-glycolic acid)^37^ and chitosan^38^ have been extensively used in NBP functionalisation. Physical routes involve passivation layers using, for example, Al_2_O_3_ (ref. 39) or capping using other 2D materials^40,41^ including graphene.^42^ Additionally, NBP have been incorporated into liposomes and gels through non-covalent functionalisation.^43,44^ Moreover, π–π stacking using pyrene has been developed for the development of self-assembled NBP loaded vesicles.^45^ The literature contains a few examples of covalent chemistry of NBP for bioconjugation. Radical addition has been carried out on NBP using diazonium salt precursors. This strategy has been used to graft 4-(6-methyl-1,3-benzothiazol-2-yl) phenylamine (a thioflavin-T derivative with high affinity to amyloid-β peptide) to prevent plaque formation in an Alzheimer's disease model.^46^ Another covalent strategy exploits the host–metal guest coordination effects.^47^ This strategy has been used to graft copper ions or copper complexes for PET imaging and photodynamic therapy.^47,48^ Additionally, P–C can be formed using halogenoalkane substrates through nucleophilic substitution.^49^ Overall, due to the rich carbon chemistry, GO remains one of the most versatile nanomaterials, facilitating different multi-functionalisation strategies. Research in covalent modification of NBP has only been developed recently, so we are expecting new synthetic strategies to come soon. In particular, the use of orthogonal reactions for multi-functionalisation has not been explored yet. For instance, esterification of phosphate groups present on NBP flakes could be a valuable covalent approach to follow.

## 3 Biodegradation by-design

**Fig. 2:**
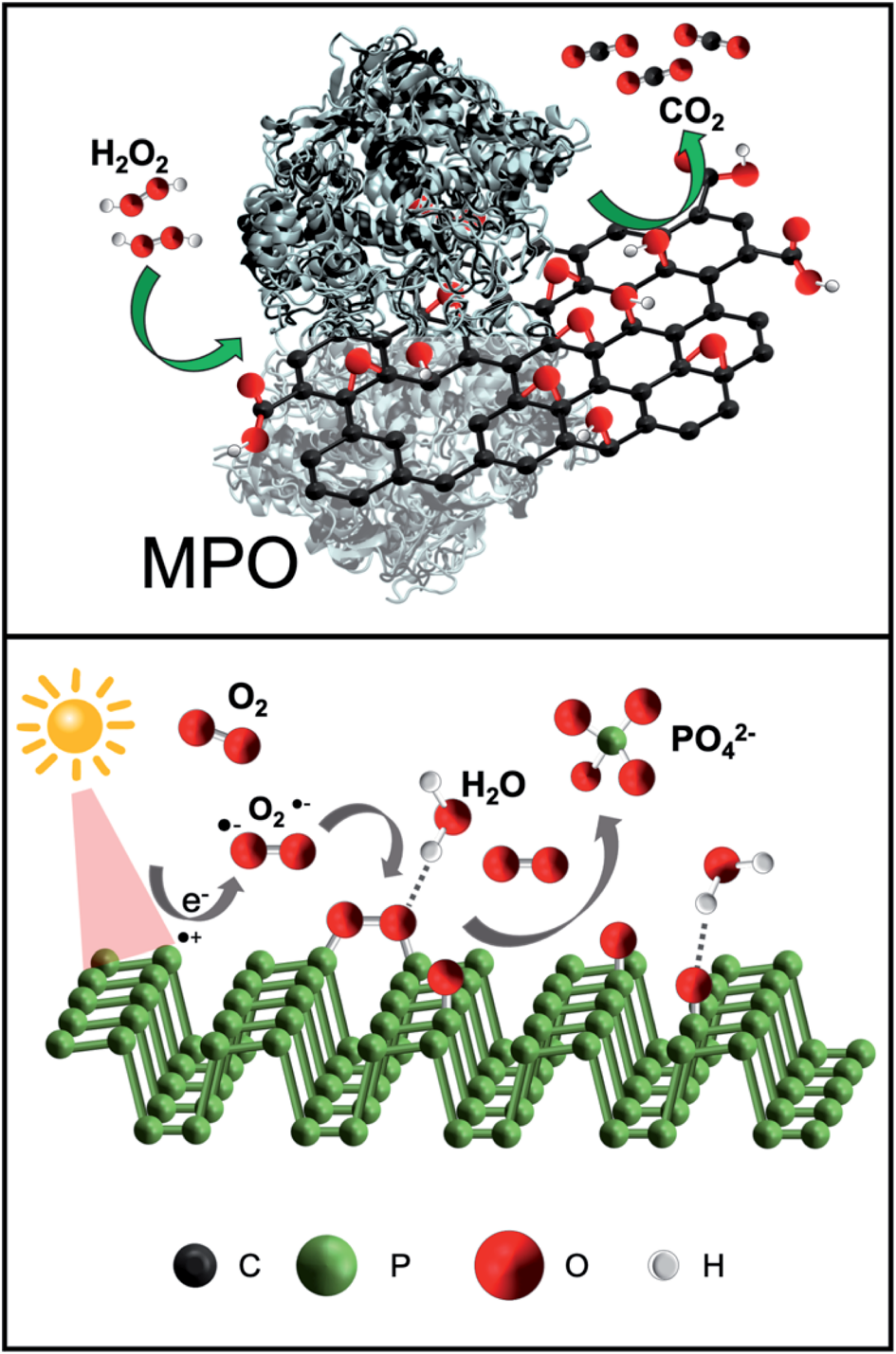
Biodegradation of GO and NBP. (Top) Scheme of the enzymatic degradation of GO. In the presence of H_2_O_2_, GO can be degraded by myeloperoxidase (MPO). (Bottom) Photodegradation mechanism of NBP in the presence of oxygen, leading to ROS formation.

The great potential of 2D materials, in the development of new hybrid platforms for biomedical applications, is well known.^50–56^ However, the elimination of nanomaterials such as NBP and GO from the body is mandatory for their possible safe and clinical use. This is the reason why the biodegradability of GO has been widely investigated in the last years.^57–59^ Although carbon nanomaterials were once considered structurally persistent, it was later demonstrated that oxidative enzymes such as peroxidases (*i.e.* myeloperoxidase, eosinophil peroxidase, *etc.*), which are secreted by neutrophils and eosinophils, are able to catalyse the degradation of carbon-based materials,^59^ including the graphene-related ones.^60^ The mechanism for the biodegradation is based on the peroxidase catalytic cycle of the enzymes in the presence of hydrogen peroxide (Fig. 2, top).^59,61^ Furthermore, although peroxidases mediated oxidation is the main process of biodegradation of carbon nanomaterials, other biodegradation pathways have been recently reported.^62^

Fadeel *et al.* carefully reviewed the role of physicochemical properties, including the number of layers, the lateral dimension, and the C/O ratio, for the safety of graphene-based materials in humans and the environment.^63^ In the specific case of GO, the functional groups that are present on its surface allow a better dispersibility of the nanomaterial, which is positive for a faster degradation rate.^64^ Furthermore, tailored functionalisation enables specific applications of the final hybrid (*e.g.* targeting and imaging). One of the most interesting biomedical applications of GO is the development of site-directed (targeted) systems for drug delivery applications, since its surface chemistry allows the grafting of a large number of ligands with specific targeting capabilities.^65,66^ In our recent work, we functionalised GO sheets with the chemotactic peptide *N*-formyl-methionyl-leucyl-phenylalanine, which is known to interact with the formyl peptide receptor expressed in different cancer cells.^67^ The engineered multifunctional GO material loaded with doxorubicin (an anticancer drug) was able to deliver the drug into cancer cells in a targeted way, also displaying an improved biodegradation ability in the presence of human myeloperoxidase under physiological conditions. In addition, peptide-functionalised GO was able to induce neutrophil degranulation with subsequent degradation, being the first study showing inducible neutrophil degradation by the nanomaterial itself with no prior activation of the cells.

Two-dimensional NBP, like GO, provides excellent potential for applications in biomedicine in terms of drug delivery and phototherapy. The already mentioned lack of stability of BP in an aqueous solution could compromise the biological applications of NBP or it could be a major benefit of its use because it can be readily degraded into biocompatible ions.^68^ The NBP degradation mechanism is strongly determined by the environmental conditions. Upon light irradiation, the surface of the NBP layer is transformed to oxidised phosphorus due to the combination of O_2_˙^−^ with NBP (Fig. 2, bottom). Secondly, oxidised phosphorus reacts with water, resulting in phosphate ions. In other words, the exposure of NBP to ambient light results in the formation of reactive oxygen species (ROS) on the surface that can degrade the material.^69^ The role of light on the formation of ROS is still under investigation together with its interplay with oxygen.^70^ It has been previously reported that ionic liquids allow BP to remain stable for several weeks, as they act as effective quenchers of ROS on the surface of the nanomaterial, indicating that ROS are key factors in the fast degradation of NBP.^31^

It has been suggested that the anticancer activity of NBP derives from the accelerated intracellular degradation of the nanomaterial due to the strong oxidative stress in cancer cells. This could result in drastic changes in the intracellular ionic equilibrium. In contrast, NBP would not be dangerous for normal cells since these have a milder intracellular environment.^71^ However, further investigation is still required to shed light on the selectivity of NBP to induce toxicity. In this context, Wang *et al.* described ultrathin black phosphorus nanosheets for efficient ROS generation, more specifically singlet oxygen (^1^O_2_), upon light irradiation.^68^ The authors demonstrated the therapeutic potential against cancer due to the photo-degradable ability of BP; the fresh black phosphorus nanosheets could be fully degraded to phosphate ions, or other P_*x*_O_*y*_ species under intermittent light irradiation in water. This marriage of excellent photothermal capacity with rapid degradability, which are the two main characteristics, makes NBP an attractive candidate for clinical translation.

As it has been previously described in Section 2, other different processes have been explored to protect NBP, namely physical and chemical strategies.^72^ Polymer capping such as poly(lactic-*co*-glycolic acid)^73^ or polyethylenimine (PEI)^74^ has also been used to prolong the shelf life of the material and for the design of efficient phototherapeutic/drug delivery NBP-based platforms. In conclusion, while the surface of GO is chemically modified to enhance its biodegradability,^58,75^ NBP is normally functionalised to slow down its degradation rate, making it a more stable material.^72^ Additionally, in the case of NBP, biodegradation can be triggered through light stimuli for the on-demand production of ROS, a strategy that has several benefits in cancer treatment and other diseases.

## 4 Phototherapy and immunotherapy

Phototherapy for cancer treatment has unique advantages, such as high therapeutic efficiency, minimal invasiveness, good tumour targeting, few side effects, low systemic toxicity, and little multidrug resistance compared with traditional tumour therapy drugs.^1^ 2D-materials like GO or NBP have shown promising results in addressing the challenges of phototherapy, especially those related to improving tumour targeting to avoid thermal damage of normal tissues and integrating with other treatment options like immunotherapy to reduce the possibilities of recurrence and metastasis with insufficient phototherapy alone. In general, they have been applied in photothermal therapy (PTT) and/or photodynamic therapy (PDT). Photothermal therapy (PTT) employs the photo-absorbing capacity of the material to generate heat from light, leading to the thermal ablation of cancer cells and subsequent cell death. Different from PTT, photodynamic therapy (PDT) uses singlet oxygen or other ROS, generated from the material used as a photosensitiser (PS) under light exposure, to kill cancer cells.^76^

Since 2012, the photothermal activity of GO has been studied and described.^77^ Interestingly, it has been found that NIR absorption of GO is size-dependent, although this mechanism is not yet fully understood.^78^ Upon reduction, the produced rGO showed a 3–4 fold increase of NIR absorption at 808 nm compared to un-reduced GO–PEG, and they showed its outstanding behaviour as a photothermal agent that enabled highly efficient *in vivo* tumour ablation.^77^ NBP is a potent photothermal agent.^79^ The size-dependent photothermal ability of NBP has also been systematically investigated, showing that the larger the size evaluated (size and thickness of 394 ± 75 nm and 15–18 nm) the better the photothermal performance under NIR laser irradiation.^80^ These results have also been confirmed *in vitro* on human breast cancer cells (MCF-7).^80^ For improving the stability and therapeutic efficacy of both GO and NBP, different biocompatible polymers have been used in PTT studies *in vitro* and *in vivo*. Coating with PEG,^77,81^ encapsulation in PLGA nanoparticles,^82^ or the use of hydrogels^83,84^ are very common strategies described in the literature.

The suitability of GO for PDT applications has been widely studied. GO has *per se* PDT activity, being able to generate radical species upon visible light irradiation.^85^ However, the low efficacy together with the high energy required for irradiation make this approach less interesting for cancer therapy. In most of the cases, GO is used for the delivery of PSs in cancer tissues. Functionalisation with PSs and chemotherapeutic drugs is performed through non-covalent chemistry exploiting electrostatic or hydrophobic interactions and π–π stacking capability of the GO amphiphilic surface. For example, GO has been used to improve some of the conventional limitations showed by photosensitisers such as low solubility, poor delivery efficiency, and inability to penetrate into deeper regions of the skin.^86,87^ Upon incorporation of photosensitisers on a GO platform in a formulation with high aqueous solubility and good colloidal stability, the stability and bioavailability of the photosensitiser is improved and, in consequence, the photodynamic treatment is enhanced. In this area, NBP is a rising star and has attracted enormous attention in recent years. Thanks to its photodegradation mechanism, NBP can be used directly as a PS, generating a high quantity of ROS under a xenon lamp or 660 nm laser.^68^ The mechanism of specific targeting by BP-related materials is not yet well understood but the mitochondria have already been identified as one of the most susceptible target organelles.^88^ In a different work, Liu *et al.* designed a biodegradable porous platform based on a Zr(iv)-based porphyrinic coordination network and BP.^89^ The nanomaterial was suitable for photodynamic therapy due to the elevated amounts of ROS produced and its ability to alter the essential regulators of cell survival. In order to reach deeper tissues and generate a larger amount of ROS, NBP nanocomposites have been engineered with different integrated up-conversion nanoparticles.^90,91^ In these cases, the nanoparticles distributed on the surface of the nanomaterial can undergo efficient energy transfer, mediated by 808 nm laser, to the NBP *via* the FRET process, promoting NBP to generate ROS. Similar multifunctional platforms using GO have been developed by covalently grafting up-conversion nanoparticles to its surface to use them as an imaging probe and dual PTT/PDT agent.^92^

**Fig. 3:**
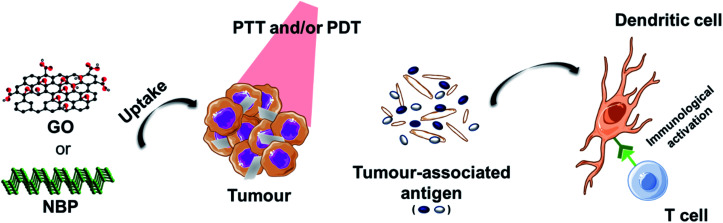
Scheme of immunotherapy activated by phototherapy. After incubation with the material, tumour cells are irradiated. Phototherapy induces damage in tumour cells producing tumour-associated antigens that are recognised by sentinel cells, thus stimulating an innate and/or adaptive immune response.

Another interesting approach that has been exploited is the enhancement of phototherapy by immunotherapy (Fig. 3). Deep or metastatic tumours are very difficult to treat by phototherapy alone. It has been observed that single PTT weakly activates the immune system due to different factors. For example, the increase of the temperature above 45 °C, necessary for tumour ablation, has shown to inhibit the activation of the immune response in the tumour microenvironment by heat-induced damage to the vasculature, suppression of chemokines and cytokines, and the temperature-induced stress in stromal and tumour cells.^1^ In order to reverse the immunosuppression induced by the PTT, different strategies involving the decoration of these nanomaterials with immune checkpoint blockers have been used. Yu *et al.* functionalised GO with the tumour integrin αvβ6-targeting peptide and the photosensitiser HPPH (2-[1-hexyloxyethyl]-2-devinyl pyropheophorbide) for PDT-enhanced immunotherapy.^93^ It is important to mention that the most common response to PTT alone for tumour ablation is necrosis.^94^ In this case, the authors exploited the combination of laser irradiation with necrotic tumour cells that favourably activated dendritic cells and induced the infiltration of cytotoxic CD8^+^ T lymphocytes into the tumours, thus preventing tumour growth and lung metastasis.^93^ Ye *et al.* prepared a hydrogel matrix containing NBP, a granulocyte-macrophage colony-stimulating factor (GM-CSF) and lipopolysaccharide (LPS) for the immunotherapy of postsurgical cancer to prevent tumour recurrence and cancer cell metastasis.^95^ After removing the primary tumour using this nanocomposite under laser irradiation, the authors observed the recruitment of dendritic cells into the area, thanks to the presence of LPS as an immunoadjuvant, and the combination with PD-1 antibody significantly enhanced tumour-specific CD8^+^ T cell elimination of the surgical residual and lung metastatic tumour.

In summary, these strategies, mutually complementing chemotherapy/phototherapy with immunotherapy, considerably suppress the proliferative capacity of the tumours and eradicates their relapse potential. However, the use of GO and NBP as functional materials still shows some limitations. 2D-materials have been profoundly analysed as carriers having a large surface area that favours drug loading. However, what occurs inside the body after the administration has not been adequately noticed. The information regarding the actual delivery of the payload is still scarce.^96^ Besides, uncertain doses of combination therapy agents, encountering cells from the reticuloendothelial system in the bloodstream, and biodegradability issues have resulted in a lack of sufficiently positive outcomes to move to the clinical stage.^1^ The clinical application of this strategy is also influenced by the tumour model chosen. Small and more superficial tumours can be completely ablated through single PTT without inducing an immune response. For large tumours, a high temperature is needed and consequently limited immune activation can be achieved. Such circumstances warrant the combination of phototherapy and immunotherapy; however, the clinical translation remains challenging.

## 5 Conclusions and perspectives

In this work, we have reported a direct comparison between GO and NBP applications for cancer therapy. In general, GO has been much more studied and many of the “lessons learned” for this nanomaterial could also be applied to NBP. From a strictly synthetic point of view, GO is much easier and more cost effective to prepare, even in the light of new studies reporting the production of NBP from cheap P precursors. Regarding the surface chemistry, NBP has been minimally explored and approaches focused on the apical phosphate groups have not yet been reported. Regarding shelf-life, GO surface derivatisation is necessary to enhance the biodegradation process, but for NBP, surface coating is necessary to retard the material decomposition. Additionally, NBP shows an interesting light triggered ROS producing a photodegradation mechanism, making it active both in PDT and PTT. GO, in contrast, is a good probe for PTT but not in PDT. However, it has not yet reached the clinical stages. So far, the only plasmonic nanophotothermal agent in clinical trials for the treatment of atherosclerosis is based on silica–gold and silica–gold iron-bearing nanoparticles (NCT01270139). Finally, both materials have shown interesting anticancer activity in drug delivery, phototherapy, and in stimulating the immune system by promoting innate and/or adaptive immune responses. However, even when the trend in recent years shows a rapidly growing interest in GO and NBP for cancer PDT,^97^ none of the 610 clinical trials^98^ based on PDT, at the time of writing this article, involve the use of graphene oxide. Furthermore, research using NBP is in an early stage compared with the advances reported with GO. Nevertheless, the investigation of these two candidates for PDT, despite promising results obtained *in vitro* and *in vivo*, still requires further efforts for their clinical translation. We believe that there is room for improvement, especially focusing on the homogenisation of the synthetic methods and on the standardisation of the biological test reported.

## Conflicts of interest

There are no conflicts to declare.

## Supplementary Material

## References

[cit1] Xie Z., Fan T., An J., Choi W., Duo Y., Ge Y., Zhang B., Nie G., Xie N., Zheng T., Chen Y., Zhang H., Kim J. S. S. (2020). Chem. Soc. Rev..

[cit2] Reina G., González-Domínguez J. M., Criado A., Vázquez E., Bianco A., Prato M. (2017). Chem. Soc. Rev..

[cit3] Qin L., Jiang S., He H., Ling G., Zhang P. (2020). J. Controlled Release.

[cit4] Nishina Y., Eigler S. (2020). Nanoscale.

[cit5] Morimoto N., Suzuki H., Takeuchi Y., Kawaguchi S., Kunisu M., Bielawski C. W., Nishina Y. (2017). Chem. Mater..

[cit6] Eigler S., Hirsch A. (2017). Phys. Sci. Rev..

[cit7] Eigler S. (2016). Chem.–Eur. J..

[cit8] Campéon B. D. L., Akada M., Ahmad M. S., Nishikawa Y., Gotoh K., Nishina Y. (2020). Carbon.

[cit9] Ou W., Byeon J. H., Thapa R. K., Ku S. K., Yong C. S., Kim J. O. (2020). ACS Nano.

[cit10] Zhang Y., Dong N., Tao H., Yan C., Huang J., Liu T., Robertson A. W., Texter J., Wang J., Sun Z. (2017). Chem. Mater..

[cit11] Poudel B. K., Hwang J., Ku S. K., Kim J. O., Byeon J. H. (2018). NPG Asia Mater..

[cit12] Sun C., Wen L., Zeng J., Wang Y., Sun Q., Deng L., Zhao C., Li Z. (2016). Biomaterials.

[cit13] Liu F., Jang M. H., Ha H. D., Kim J. H., Cho Y. H., Seo T. S. (2013). Adv. Mater..

[cit14] Sun A., Mu L., Hu X. (2017). ACS Appl. Mater. Interfaces.

[cit15] Hwang J., Le A. D. D., Trinh C. T., Le Q. T., Lee K. G., Kim J. (2021). Sens. Actuators, A.

[cit16] Yu W., Sisi L., Haiyan Y., Jie L. (2020). RSC Adv..

[cit17] Kasprzak A., Zuchowska A., Poplawska M. (2018). Beilstein J. Org. Chem..

[cit18] Vacchi I. A., Spinato C., Raya J., Bianco A., Ménard-Moyon C. (2016). Nanoscale.

[cit19] Guo S., Nishina Y., Bianco A., Ménard-Moyon C. (2020). Angew. Chem., Int. Ed..

[cit20] Vuppaladadium S. S. R., Agarwal T., Kulanthaivel S., Mohanty B., Barik C. S., Maiti T. K., Pal S., Pal K., Banerjee I. (2020). Mater. Sci. Eng., C.

[cit21] Guo S., Raya J., Ji D., Nishina Y., Ménard-Moyon C., Bianco A. (2020). Nanoscale Adv..

[cit22] He D., He X., Wang K., Zou Z., Yang X., Li X. (2014). Langmuir.

[cit23] Martín C., Martín-Pacheco A., Naranjo A., Criado A., Merino S., Díez-Barra E., Herrero M. A., Vázquez E. (2019). Nanoscale.

[cit24] Yi J., Choe G., Park J., Lee J. Y. (2020). Polym. J..

[cit25] Guilbaud-Chéreau C., Dinesh B., Schurhammer R., Collin D., Bianco A., Ménard-Moyon C. (2019). ACS Appl. Mater. Interfaces.

[cit26] Thurakkal S., Zhang X. (2020). Adv. Sci..

[cit27] Xu C., Xu Y., Yang M., Chang Y., Nie A., Liu Z., Wang J., Luo Z. (2020). Adv. Funct. Mater..

[cit28] Liu Y., Gao P., Zhang T., Zhu X., Zhang M., Chen M., Du P., Wang G., Ji H., Yang J., Yang S. (2019). Angew. Chem., Int. Ed..

[cit29] Ryder C. R., Wood J. D., Wells S. A., Yang Y., Jariwala D., Marks T. J., Schatz G. C., Hersam M. C. (2016). Nat. Chem..

[cit30] Artel V., Guo Q., Cohen H., Gasper R., Ramasubramaniam A., Xia F., Naveh D. (2017). npj 2D Mater. Appl..

[cit31] Walia S., Balendhran S., Ahmed T., Singh M., El-Badawi C., Brennan M. D., Weerathunge P., Karim M. N., Rahman F., Rassell A., Duckworth J., Ramanathan R., Collis G. E., Lobo C. J., Toth M., Kotsakidis J. C., Weber B., Fuhrer M., Dominguez-Vera J. M., Spencer M. J. S., Aharonovich I., Sriram S., Bhaskaran M., Bansal V. (2017). Adv. Mater..

[cit32] Shao J., Xie H., Huang H., Li Z., Sun Z., Xu Y., Xiao Q., Yu X.-F., Zhao Y., Zhang H., Wang H., Chu P. K. (2016). Nat. Commun..

[cit33] Chen H., Liu Z., Wei B., Huang J., You X., Zhang J., Yuan Z., Tang Z., Guo Z., Wu J. (2021). Bioact. Mater..

[cit34] Xie Z., Peng M., Lu R., Meng X., Liang W., Li Z., Qiu M., Zhang B., Nie G., Xie N., Zhang H., Prasad P. N. (2020). Light: Sci. Appl..

[cit35] Zhang Q., Wang W., Zhang M., Wu F., Zheng T., Sheng B., Liu Y., Shen J., Zhou N., Sun Y. (2020). Chem. Eng. J..

[cit36] Yang X., Wang D., Zhu J., Xue L., Ou C., Wang W., Lu M., Song X., Dong X. (2019). Chem. Sci..

[cit37] Tong L., Liao Q., Zhao Y., Huang H., Gao A., Zhang W., Gao X., Wei W., Guan M., Chu P. K., Wang H. (2019). Biomaterials.

[cit38] Pan W., Dai C., Li Y., Yin Y., Gong L., achwa Machuki J. O., Yang Y., Qiu S., Guo K., Gao F. (2020). Biomaterials.

[cit39] Illarionov Y. Y., Waltl M., Rzepa G., Knobloch T., Kim J.-S., Akinwande D., Grasser T. (2017). npj 2D Mater. Appl..

[cit40] Gamage S., Fali A., Aghamiri N., Yang L., Ye P. D., Abate Y. (2017). Nanotechnology.

[cit41] Son Y., Kozawa D., Liu A. T., Koman V. B., Wang Q. H., Strano M. S. (2017). 2D Mater..

[cit42] Kim J., Baek S. K., Kim K. S., Chang Y. J., Choi E. J. (2016). Curr. Appl. Phys..

[cit43] Zhang L., Wang Y., Wang J., Wang Y., Chen A., Wang C., Mo W., Li Y., Yuan Q., Zhang Y. (2019). ACS Appl. Mater. Interfaces.

[cit44] Cheng L., Cai Z., Zhao J., Wang F., Lu M., Deng L., Cui W. (2020). Bioact. Mater..

[cit45] Li Z., Hu Y., Fu Q., Liu Y., Wang J., Song J., Yang H. (2020). Adv. Funct. Mater..

[cit46] Li Y., Du Z., Liu X., Ma M., Yu D., Lu Y., Ren J., Qu X. (2019). Small.

[cit47] Zhang D., Lin Z., Lan S., Sun H., Zeng Y., Liu X. (2019). Mater. Chem. Front..

[cit48] Hu K., Xie L., Zhang Y., Hanyu M., Yang Z., Nagatsu K., Suzuki H., Ouyang J., Ji X., Wei J., Xu H., Farokhzad O. C., Liang S. H., Wang L., Tao W., Zhang M. R. (2020). Nat. Commun..

[cit49] Sofer Z., Luxa J., Bouša D., Sedmidubský D., Lazar P., Hartman T., Hardtdegen H., Pumera M. (2017). Angew. Chem., Int. Ed..

[cit50] Radhapyari K. , Datta S., Dutta S., Jadon N. and Khan R., in Two-Dimensional Nanostructures for Biomedical Technology: A Bridge Between Material Science and Bioengineering, Elsevier, 2019, pp. 101–135

[cit51] Dasari Shareena T. P., McShan D., Dasmahapatra A. K., Tchounwou P. B. (2018). Nano-Micro Lett..

[cit52] Zong S., Wang L., Yang Z., Wang H., Wang Z., Cui Y. (2019). ACS Appl. Mater. Interfaces.

[cit53] Rohaizad N., Mayorga-Martinez C. C., Fojtů M., Latiff N. M., Pumera M. (2021). Chem. Soc. Rev..

[cit54] Bolotsky A., Butler D., Dong C., Gerace K., Glavin N. R., Muratore C., Robinson J. A., Ebrahimi A. (2019). ACS Nano.

[cit55] Tao W., Kong N., Ji X., Zhang Y., Sharma A., Ouyang J., Qi B., Wang J., Xie N., Kang C., Zhang H., Farokhzad O. C., Kim J. S. (2019). Chem. Soc. Rev..

[cit56] Ghosal K., Sarkar K. (2018). ACS Biomater. Sci. Eng..

[cit57] Martín C., Kostarelos K., Prato M., Bianco A. (2019). Chem. Commun..

[cit58] Martín C., Jun G., Schurhammer R., Reina G., Chen P., Bianco A., Ménard-Moyon C. (2019). Small.

[cit59] Kotchey G. P., Hasan S. A., Kapralov A. A., Ha S. H., Kim K., Shvedova A. A., Kagan V. E., Star A. (2012). Acc. Chem. Res..

[cit60] Kotchey G. P., Allen B. L., Vedala H., Yanamala N., Kapralov A. A., Tyurina Y. Y., Klein-Seetharaman J., Kagan V. E., Star A. (2011). ACS Nano.

[cit61] Kurapati R., Martìn C., Palermo V., Nishina Y., Bianco A. (2021). Faraday Discuss..

[cit62] Peng G., Montenegro M. F., Ntola C. N. M., Vranic S., Kostarelos K., Vogt C., Toprak M. S., Duan T., Leifer K., Bräutigam L., Lundberg J. O., Fadeel B. (2020). Nanoscale.

[cit63] Fadeel B., Bussy C., Merino S., Vázquez E., Flahaut E., Mouchet F., Evariste L., Gauthier L., Koivisto A. J., Vogel U., Martín C., Delogu L. G., Buerki-Thurnherr T., Wick P., Beloin-Saint-Pierre D., Hischier R., Pelin M., Candotto Carniel F., Tretiach M., Cesca F., Benfenati F., Scaini D., Ballerini L., Kostarelos K., Prato M., Bianco A. (2018). ACS Nano.

[cit64] Kurapati R., Russier J., Squillaci M. A., Treossi E., Ménard-Moyon C., Del Rio-Castillo A. E., Vazquez E., Samorì P., Palermo V., Bianco A. (2015). Small.

[cit65] Karki N., Tiwari H., Tewari C., Rana A., Pandey N., Basak S., Sahoo N. G. (2020). J. Mater. Chem. B.

[cit66] Liu C.-C., Zhao J.-J., Zhang R., Li H., Chen B., Zhang L.-L., Yang H. (2017). Am. J. Transl. Res..

[cit67] Martín C., Ruiz A., Keshavan S., Reina G., Murera D., Nishina Y., Fadeel B., Bianco A. (2019). Adv. Funct. Mater..

[cit68] Wang H., Yang X., Shao W., Chen S., Xie J., Zhang X., Wang J., Xie Y. (2015). J. Am. Chem. Soc..

[cit69] Zhou Q., Chen Q., Tong Y., Wang J. (2016). Angew. Chem..

[cit70] Ahmed T., Balendhran S., Karim M. N., Mayes E. L. H., Field M. R., Ramanathan R., Singh M., Bansal V., Sriram S., Bhaskaran M., Walia S. (2017). npj 2D Mater. Appl..

[cit71] Zhou W., Pan T., Cui H., Zhao Z., Chu P. K., Yu X. (2018). Angew. Chem..

[cit72] Kuriakose S., Ahmed T., Balendhran S., Bansal V., Sriram S., Bhaskaran M., Walia S. (2018). 2D Mater..

[cit73] Xu X., Jiang Y., Wang M., Wang H., Lu C., Yang H. (2020). Part. Part. Syst. Charact..

[cit74] Chen L., Chen C., Chen W., Li K., Chen X., Tang X., Xie G., Luo X., Wang X., Liang H., Yu S. (2018). ACS Appl. Mater. Interfaces.

[cit75] Kurapati R., Bonachera F., Russier J., Sureshbabu A. R., Ménard-Moyon C., Kostarelos K., Bianco A. (2017). 2D Mater..

[cit76] Choi J. R., Yong K. W., Choi J. Y., Nilghaz A., Lin Y., Xu J., Lu X. (2018). Theranostics.

[cit77] Yang K., Wan J., Zhang S., Tian B., Zhang Y., Liu Z. (2012). Biomaterials.

[cit78] Liu J., Cui L., Losic D. (2013). Acta Biomater..

[cit79] Ouyang J., Feng C., Zhang X., Kong N., Tao W. (2021). Acc. Mater. Res..

[cit80] Fu H., Li Z., Xie H., Sun Z., Wang B., Huang H., Han G., Wang H., Chu P. K., Yu X.-F. (2017). RSC Adv..

[cit81] Sun Z., Xie H., Tang S., Yu X.-F., Guo Z., Shao J., Zhang H., Huang H., Wang H., Chu P. K. (2015). Angew. Chem..

[cit82] Mohammadi Gazestani A., Khoei S., Khoee S., Emamgholizadeh Minaei S., Motevalian M. (2018). Artif. Cells, Nanomed., Biotechnol..

[cit83] Zhu X., Zhang Y., Huang H., Zhang H., Hou L., Zhang Z. (2016). J. Biomater. Appl..

[cit84] Phan L. M. T., Vo T. A. T., Hoang T. X., Cho S. (2021). Nanomaterials.

[cit85] Hu X., Mu L., Wen J., Zhou Q. (2012). Carbon.

[cit86] Detty M. R., Gibson S. L., Wagner S. J. (2004). J. Med. Chem..

[cit87] Huang Z. (2005). Technol. Cancer Res. Treat..

[cit88] Pan T., Fu W., Xin H., Geng S., Li Z., Cui H., Zhang Y., Chu P. K., Zhou W., Yu X. (2020). Adv. Funct. Mater..

[cit89] Liu M.-D., Yu Y., Guo D.-K., Wang S.-B., Li C.-X., Gao F., Zhang C., Xie B.-R., Zhong Z., Zhang X.-Z. (2020). Nanoscale.

[cit90] Xie X., Gao N., Deng R., Sun Q., Xu Q.-H., Liu X. (2013). J. Am. Chem. Soc..

[cit91] Lv R., Yang D., Yang P., Xu J., He F., Gai S., Li C., Dai Y., Yang G., Lin J. (2016). Chem. Mater..

[cit92] Wang Y., Wang H., Liu D., Song S., Wang X., Zhang H. (2013). Biomaterials.

[cit93] Yu X., Gao D., Gao L., Lai J., Zhang C., Zhao Y., Zhong L., Jia B., Wang F., Chen X., Liu Z. (2017). ACS Nano.

[cit94] Pérez-Hernández M., del Pino P., Mitchell S. G., Moros M., Stepien G., Pelaz B., Parak W. J., Gálvez E. M., Pardo J., de la Fuente J. M. (2015). ACS Nano.

[cit95] Ye X., Liang X., Chen Q., Miao Q., Chen X., Zhang X., Mei L. (2019). ACS Nano.

[cit96] Hoseini-Ghahfarokhi M., Mirkiani S., Mozaffari N., Abdolahi Sadatlu M. A., Ghasemi A., Abbaspour S., Akbarian M., Farjadain F., Karimi M. (2020). Int. J. Nanomed..

[cit97] Gazzi A., Fusco L., Khan A., Bedognetti D., Zavan B., Vitale F., Yilmazer A., Delogu L. G. (2019). Front. Bioeng. Biotechnol..

[cit98] U.S. National Library of Medicine , Clinicaltrials.gov, https://clinicaltrials.gov/ct2/home

